# Genome Sequences of Five Nonvirulent *Listeria monocytogenes* Serovar 4 Strains

**DOI:** 10.1128/genomeA.00179-16

**Published:** 2016-03-31

**Authors:** Eric Sumrall, Jochen Klumpp, Yang Shen, Martin J. Loessner

**Affiliations:** Institute of Food, Nutrition and Health, ETH Zürich, Zürich, Switzerland

## Abstract

We present the complete genome sequences of five nonpathogenic *Listeria monocytogenes* serovar 4 strains: WSLC 1018 (4e), 1019 (4c), 1020 (4a), 1033 (4d), and 1047 (4d). These sequences may help to uncover genes involved in the synthesis of the serovar antigens—phenotypic determinants of virulence deemed clinically relevant.

## GENOME ANNOUNCEMENT

*Listeria monocytogenes* is a Gram-positive, facultative, intracellular bacterium and one of the most common food-borne pathogens, being able to cause serious infection in susceptible individuals and frequently leading to fatality (1). It is therefore of paramount importance to discover and develop better methods for biocontrol, and to further elucidate virulence markers in this bacterium.

*L. monocytogenes* serovar 4 contains multiple variants, only one of which (serovar 4b) is known to cause infection in humans, and is one of the most common pathogenic subtypes (1). In general, strains belonging to serovar 4 are susceptible to bacteriophage infection, but curiously, seldom contain intact prophages (2). It has been suggested that this can be attributed to differences in cell-wall composition (2). Additionally, the fundamental differences in cell-wall structure between the virulent and nonvirulent strains of *L. monocytogenes* within serovar 4 are poorly understood, particularly with respect to the cell-wall networks defining the outer antigens that correspond to the serovar (3). Interestingly, specific molecules associated with the cell surface molecules can be involved in both phage binding (4) and serovar determination in *L. monocytogenes* (5, 6), meaning that phage-resistant strains can be used as tools to study cell-wall composition. Given this fact, the ultimate goal of this sequencing project is to explain differences in cell-wall biosynthesis genes between isolates within serovar 4, which is also expected to lead to a better understanding of the genetic and phenotypic basis of cell wall–mediated phage resistance. This will be done in conjunction with the recently sequenced 4b strain, WSLC 1042 (7). As the genetic foundation of cell-wall composition in *L. monocytogenes* seems to be quite diversified, but is not yet fully understood, sequencing for the purpose of *de novo* assembly is required.

Representative strains of the serovar 4 group were grown at 30°C in half-concentrated brain heart infusion (BHI) medium under aerobic conditions. Whole-genomic DNA was isolated and subjected to single-molecule real-time sequencing on a Pacific Biosciences RS2 device (10-kb insert library, P6/C4 chemistry) at the Functional Genomics Center Zurich (Zurich, Switzerland). Sequencing resulted in 96,820 reads, with a mean read length of 13,116 bp for WSLC 1018; 92,890 reads with a 10,781-bp mean length for WSLC 1019; 113,785 reads with a mean length of 12,784 bp for WSLC 1020; 98,676 reads with a mean length of 13,441 bp for WSLC 1033, and 100,720 reads with a mean length of 13,319 bp for WSLC 1047. Genomes were assembled *de novo* using SMRT Analysis version 2.3 software and the HGAP3 algorithm. Final assembly produced single linear contigs with lengths of 2,942,510 bp for WSLC 1018 (265-fold coverage), 2,839,870 bp for WSLC 1019 (211-fold coverage), 2,842,039 bp for WSLC 1020 (230-fold coverage), 2,950,820 bp (278-fold coverage) for WSLC 1033, and 2,950,749 bp (299-fold coverage) for WSLC 1047. Finally, annotation of the genomes was performed by the NCBI Prokaryotic Genome Automatic Pipeline, which determined that strains 1018, 1019, 1020, 1033, and 1047 each encoded 2,920, 2,801, 2,808, 2,920, and 2,918 genes, respectively.

### Nucleotide sequence accession numbers.

The complete genome sequences have been deposited in GenBank under the accession numbers CP013285, CP013286, CP013287, CP013288, and CP013289.
